# A Systemically Administered Unconjugated Antisense Oligonucleotide Targeting DUX4 Improves Muscular Injury and Motor Function in FSHD Model Mice

**DOI:** 10.3390/biomedicines11092339

**Published:** 2023-08-22

**Authors:** Tetsuhiro Kakimoto, Akira Ogasawara, Kiyoshi Ishikawa, Takashi Kurita, Kumiko Yoshida, Shuichi Harada, Taeko Nonaka, Yoshimi Inoue, Keiko Uchida, Takashi Tateoka, Tetsuya Ohta, Shinji Kumagai, Takashi Sasaki, Hajime Aihara

**Affiliations:** Sohyaku. Innovative Research Division, Mitsubishi Tanabe Pharma Corporation, 2-26-1 Muraoka-Higashi, Fujisawa-shi, Kanagawa 251-8555, Japan

**Keywords:** facioscapulohumeral muscular dystrophy, antisense oligonucleotide, muscle injury, motor function, muscle force, DUX4

## Abstract

Facioscapulohumeral muscular dystrophy (FSHD), one of the most common muscular dystrophies, is caused by an abnormal expression of the DUX4 gene in skeletal muscles, resulting in muscle weakness. In this study, we investigated MT-DUX4-ASO, a novel gapmer antisense oligonucleotide (ASO). MT-DUX4-ASO decreased the expression of DUX4 and its target genes in FSHD patient-derived myoblasts. For the first time, we demonstrated that a systemically administered ASO, even without a ligand for drug delivery, could significantly improve muscle injury and motor function in the ACTA1-MCM/FLExDUX4 (DUX4-TG) mouse model of FSHD. Tamoxifen (TMX) injection transiently induces skeletal-muscle-specific DUX4 expression in DUX4-TG mice, while the skeletal muscles of TMX-untreated DUX4-TG mice have leaky DUX4 expression in a small subset of myofibers similar to those of FSHD patients. Subcutaneous 10 mg/kg of MT-DUX4-ASO at two-week intervals significantly suppressed muscular DUX4 target gene expression, histological muscle injury, and blood muscle injury marker elevation in TMX-untreated DUX4-TG mice. Notably, MT-DUX4-ASO at 10 mg/kg every other week significantly prevented the TMX-induced declines in treadmill test running speed and muscle force in DUX4-TG mice. Thus, the systemically administered unconjugated MT-DUX4-ASO suppressed disease progression in DUX4-TG mice, extending the potential of unconjugated ASOs as a promising FSHD treatment strategy.

## 1. Introduction

FSHD is the third most common hereditary muscular dystrophy, with an estimated prevalence of 1:15,000–1:20,000 [1,2]. FSHD progresses slowly and asymmetrically. The muscles of the face and shoulder are usually the first to be affected, followed by the truncal and lower extremity muscles. Muscle weakness impairs mobility, including lifting objects and ambulating [3,4]. For example, more than 20% of patients require wheelchair use [5]. These symptoms have a significant impact on FSHD patients’ lives. Nevertheless, there exists no effective disease-modifying drug for FSHD.

FSHD is caused by stochastic aberrant misexpression of the double homeobox 4 (DUX4) gene in skeletal muscles [1,2,6]. The D4Z4 macrosatellite repeat on the permissive 4qA haplotype of chromosome 4q35 is contracted to 1–10 repeat units in FSHD1 patients, accounting for 95% of FSHD, leading to epigenetic derepression of DUX4, which is normally silenced in adult somatic cells. FSHD2 is caused by chromatin modifier gene mutations, which also result in the epigenetic derepression of DUX4. FSHD1 and FSHD2 have similar clinical presentations. The pathogenic DUX4 gene is typically expressed in only a small fraction of skeletal muscle cells in FSHD patients [7]. In skeletal muscles, the causal DUX4 protein acts as a cytotoxic transcription factor, causing cell death via downstream gene expression [8].

Because of its pathophysiological similarities to DUX4-induced FSHD, ACTA1-MCM/FLExDUX4 mouse (hereafter referred to as DUX4-TG mouse) was recently established as an FSHD model, and is of increasing preclinical interest [9,10]. Even without TMX induction, leaky DUX4 is expressed in only a small subset (2–10%) of myofibers in DUX4-TG muscles [10]. This leaky DUX4 expression in TMX-untreated DUX4-TG mice recapitulates DUX4 expression in only a small fraction of muscle cells in FSHD patients [7]. These TMX-untreated DUX4-TG mice show no decline in treadmill running time [10]. TMX injection causes skeletal-muscle-specific Cre expression, which causes transgene recombination to induce a burst of DUX4 expression in mosaic patterns in 7–28% of myofibers, resulting in more exaggerated pathologic changes including motor functional decline [10]. These exaggerated pathologic changes might recapitulate the focal active lesion in FSHD patients [11,12]. The TMX-induced muscular DUX4 expression in DUX4-TG mice is transient because DUX4-expressing cells die and surrounding non-DUX4-expressing satellite cells regenerate to compensate [10]. The treadmill running time of TMX-injected DUX4-TG mice consistently showed a transient decline and recovered after the peak of motor function decline at two weeks after the TMX injection [10].

Antisense oligonucleotides that could decrease the causative DUX4 expression have been investigated as a promising treatment strategy [13,14]. Nonetheless, only a few groups have demonstrated a systemic improvement in DUX4-TG mice using ASOs with a ligand that facilitated ASO delivery to skeletal muscles [15,16,17,18]. Here, we have generated a novel gapmer ASO targeting the human DUX4 gene, MT-DUX4-ASO, with an optimized design and high in vivo activity. A gapmer ASO can induce strong RNase H-mediated target RNA degradation. Even though MT-DUX4-ASO is unconjugated with a ligand for delivery to skeletal muscles, we found that subcutaneous administration of our optimized MT-DUX4-ASO at two-week intervals improved muscle injury and motor function decline in DUX4-TG mice. MT-DUX4-ASO at as little as 10 mg/kg every other week significantly suppressed muscular DUX4 target gene expression, histological muscular injury, and blood muscular injury biomarker elevation in TMX-untreated DUX4-TG mice. A subcutaneous 10 mg/kg dose of MT-DUX4-ASO every other week also significantly limited the TMX-induced decline in motor function and muscle force in DUX4-TG mice. Furthermore, 30 mg/kg of MT-DUX4-ASO every other week almost completely prevented the TMX-induced motor function decline in DUX4-TG mice.

## 2. Materials and Methods

### 2.1. Antisense Oligonucleotides

MT-DUX4-ASO is 16 residues in length and has a full phosphorothioate backbone. The sequence is 5′-GCCtagacagcgtCGG-3′. The central ten DNA gap regions (lower cases) are flanked by the wing regions (upper cases) of three 2′-*N*-methanesulfonyl-2′-amino-locked nucleic acid (ALNA[Ms])-modified nucleotides [19] at both ends, where C is substituted by 5-methylcytosine (mC).

### 2.2. In Vitro Luciferase Reporter Assay

C2C12 cells (American Type Culture Collection, Manassas, VA, USA) were grown at 37 °C in Dulbecco’s modified Eagle’s medium (DMEM) High Glucose, HEPES (Thermo Fisher Scientific, Waltham, MA, USA) containing 10% fetal bovine serum, 50 unit/mL penicillin, and 50 μg/mL streptomycin (Thermo Fisher Scientific). C2C12 cells were seeded into the medium containing MT-DUX4-ASO with the indicated concentration. Forty-eight hours after seeding, the cells were transfected with Lipofectamine 2000 (Thermo Fisher Scientific) with the reporter plasmid encoding the luciferase gene followed by the DUX4 open reading frame sequence inserted into the 3′-untranslated regions. The cells were lysed 24 h after the transfection, and luciferase reporter activity was measured using the Dual-Glo Luciferase Assay System (Promega, Madison, WI, USA). The IC_50_ value was calculated with nonlinear regression analysis using GraphPad Prism software (version 8.4, GraphPad Software, Boston, MA, USA).

### 2.3. In Vitro Myotube Formation and Gene Expression Analysis in Patient-Derived Myoblasts

FSHD patient-derived myoblasts (GM17940, Coriell Institute, Camden, NJ, USA) were seeded in Ham’s F-10 Nutrient mixture media (Thermo Fisher Scientific) containing 1.2 mM CaCl_2_, 20% fetal bovine serum, 100 unit/mL penicillin, and 100 μg/mL streptomycin as growth medium at 37 °C. Myoblasts were treated with the indicated concentration of MT-DUX4-ASO in the differentiation medium, DMEM/F12 (Thermo Fisher Scientific) supplemented with 5 μg/mL Insulin-Transferrin-Selenium Premix Universal Culture Supplement (Corning, Corning, NY, USA), 5% fetal bovine serum, 100 unit/mL penicillin, 100 μg/mL streptomycin, 0.1% bovine serum albumin, and 1% glutamine 24 h after seeding. Seven days after differentiation, the cells were lysed for gene expression analysis, and total RNA was extracted using the Maxwell RSC simply RNA Tissue Kit (Promega). First-strand cDNA was synthesized using ReverTra Ace qPCR RT Master Mix (Toyobo, Osaka, Japan). To quantify extremely low DUX4 expression, real-time PCR was carried out on an Applied Biosystems 7500 Fast Real-Time PCR System (Thermo Fisher Scientific) using KOD SYBR qPCR Mix (Toyobo). The following primers were used—DUX4-F: 5′-CCCAGGTACCAGCAGACC-3′; DUX4-R: 5′-TCCAGGAGATGTAACTCTAATCCA-3′; and GAPDH: Human Housekeeping Gene Primer Set (Takara Bio Inc., Shiga, Japan). DUX4 gene expression level was normalized to GAPDH reference gene expression level for presentation. The IC_50_ value was calculated with nonlinear regression analysis using GraphPad Prism software.

For myotube formation assay, FSHD patient-derived myoblasts were seeded in growth medium and differentiated in differentiation medium 24 h after seeding. The cells were treated with the indicated concentration of MT-DUX4-ASO seven days after differentiation. Fourteen days after differentiation, the cells were fixed with 4% paraformaldehyde and stained with anti-myosin heavy chain (MHC) mouse monoclonal antibody (R&D Systems, Minneapolis, MN, USA, Cat. No. MAB4470) followed by Alexa Fluor plus 488-conjugated secondary antibody (anti-mouse IgG goat polyclonal antibody; Thermo Fisher Scientific). Nuclei were stained with Hoechst 33342 (Dojindo, Kumamoto, Japan). An IN Cell Analyzer 2200 (GE Healthcare, Chicago, IL, USA) was used to process the images of 25 fields in each well. The MHC-positive area was quantified as the myotube area. The myogenic fusion index was calculated as the percentage of the nuclei number in MHC-positive cells relative to the total nuclei number [13,14,16]. Fourteen days after differentiation, the cells were lysed for gene expression analysis, and RNA was extracted using the Maxwell RSC simply RNA Tissue Kit. First-strand cDNA was synthesized using ReverTra Ace qPCR RT Master Mix. Real-time PCR was carried out on an Applied Biosystems 7500 Fast Real-Time PCR System using the Luna Universal Probe qPCR Master mix (New England BioLabs, Ipswich, MA, USA) and the following TaqMan probes—ZSCAN4: TaqMan assay ID Hs00537549_m1 (Thermo Fisher Scientific); MBD3L2: TaqMan assay ID Hs00544743_m1 (Thermo Fisher Scientific); TRIM43: TaqMan assay ID: Hs.PT.58.40460634 (Integrated DNA Technologies, Coralville, IA, USA); and 18S rRNA: Eukaryotic 18S rRNA Endogenous Control (Thermo Fisher Scientific). DUX4 target gene expression levels were normalized to 18S rRNA level for presentation. The IC_50_ value was calculated with nonlinear regression analysis using GraphPad Prism software.

### 2.4. Animals

Heterozygous ACTA1-MCM/FLExDUX4 [9,10,20] mice were obtained from the Jackson Laboratory (Bar Harbor, ME, USA) and bred at the Jackson Laboratory Japan (Yokohama, Japan). ACTA1-MCM mice (referred to as control mice) were investigated as a normal control. The animals were acclimatized for at least 5 days prior to the study, and were housed under a 12-h light/12-h dark cycle with free access to water and chow. Efficacy animal experiments were conducted according to institutional guidelines and were pre-approved by the Shonan Health Innovation Park Institutional Animal Care and Use Committee. A tolerability animal experiment was conducted according to institutional guidelines and was pre-approved by the Mitsubishi Tanabe Pharma Corporation Institutional Animal Care and Use Committee.

For the first and second in vivo experiments, MT-DUX4-ASO was dissolved in saline. Female DUX4-TG mice were randomly divided at 9 weeks of age. In the first in vivo experiment (Table 1), DUX4-TG mice were subcutaneously injected five times with 10 or 15 mg/kg (5 mL/kg) of MT-DUX4-ASO (*N* = 6 and 5, respectively) or vehicle (*N* = 5) at two-week intervals. To provide normal controls, four control mice were similarly injected with vehicle. Blood was collected from the abdominal vena cava under anesthesia 14 days after the last dose of MT-DUX4-ASO, and animals were sacrificed by exsanguination. Gastrocnemius muscles were collected from the euthanized animals. Female DUX4-TG mice were used because they have more severe diseases than male mice [10,15].

In the second in vivo experiment, female DUX4-TG mice were subcutaneously injected three times with 15 or 30 mg/kg (5 mL/kg) of MT-DUX4-ASO (*N* = 5 and 4, respectively) or vehicle (*N* = 4 each with and without TMX injection) at two-week intervals. On the day of the last MT-DUX4-ASO dose, 5 mg/kg of TMX (5 mL/kg) was injected intraperitoneally to induce DUX4 expression. To provide normal controls, five control mice were similarly injected with vehicle and TMX. Thirteen days after the last dose of MT-DUX4-ASO, a treadmill running test was performed. Blood was collected from the abdominal vena cava under anesthesia 17 days after the last dose of MT-DUX4-ASO, and animals were sacrificed by exsanguination. We failed to collect blood from one DUX4-TG mouse treated with 15 mg/kg of MT-DUX4-ASO due to a technical error. Gastrocnemius muscles were collected from the euthanized animals.

For the third in vivo experiment, MT-DUX4-ASO was dissolved in phosphate-buffered saline (PBS). From 9 weeks of age, male DUX4-TG mice were randomly divided and subcutaneously injected five times with 10 mg/kg (5 mL/kg) of MT-DUX4-ASO (*N* = 5) or vehicle (*N* = 5 and 6 with and without TMX injection, respectively) at two-week intervals. On the day of the last dose of MT-DUX4-ASO, 7.5 mg/kg of TMX (5 mL/kg) was injected intraperitoneally to induce DUX4 expression. A higher dose of TMX was injected to the male DUX4-TG mice compared to the female mice because male DUX4-TG mice have a milder disease than female mice [10,15]. Six control mice were also injected with vehicle and TMX to provide normal controls. Ten days after the last dose of MT-DUX4-ASO, a treadmill running test was performed. Animals were sacrificed by cervical dislocation under anesthesia thirteen or fourteen days after the last dose of MT-DUX4-ASO. Soleus muscles were immediately collected from the euthanized animals. Tibialis anterior muscles were also collected. Male mice were used in this experiment to demonstrate that MT-DUX4-ASO has an ameliorative effect on both sexes of DUX4-TG mice.

TMX injection solution was prepared as described previously [9]. TMX (Sigma-Aldrich, St. Louis, MO, USA) was dissolved in ethanol at 54 °C to make 100 mg/mL and was diluted with warmed corn oil (Sigma-Aldrich) to make a 10 mg/mL stock solution. Before injection, stock TMX solution was further diluted with corn oil to make the injectable solution.

For the in vivo tolerability experiment, MT-DUX4-ASO was dissolved in saline. From 6 weeks of age, male normal Crl:CD1 (ICR) mice obtained from Jackson Laboratory Japan were randomly divided and administered once daily for 4 days with 100 mg/kg (5 mL/kg) of MT-DUX4-ASO (*N* = 5) or vehicle (*N* = 5) intravenously. Blood was collected from the abdominal vena cava under anesthesia 3 days after the last dose of MT-DUX4-ASO, and animals were sacrificed by exsanguination. Serum fraction was separated by centrifugation of collected blood samples. Blood chemistry was measured using a Hitachi Automatic Analyzer Labospect 006 (Hitachi High-Technologies, Tokyo, Japan).

### 2.5. Gene Expression Analysis of Muscles

RNA was isolated from muscles using the Maxwell RSC simplyRNA Tissue Kit. First-strand cDNA was synthesized using SuperScript IV First-Strand Synthesis System (Thermo Fisher Scientific). To quantify extremely low DUX4 expression, real-time PCR was performed on a 7500 Fast Real-Time PCR System or StepOnePlus real-time PCR system (Thermo Fisher Scientific) using KOD SYBR qPCR Mix. The following primers were used—DUX4-F: 5′-GCGCAACCTCTCCTAGAAAC-3′; DUX4-R: 5′-AGAGCCCGGTATTCTTCCT-3′; Wfdc3: Mm.PT.58.14123465 (Integrated DNA Technologies); and Mrpl19: Mm.PT.58.45868308 (Integrated DNA Technologies). Wfdc3 gene expression was determined by real-time PCR on a StepOnePlus real-time PCR system with a Luna Universal Probe qPCR Master Mix using the TaqMan Gene Expression Assay (Wfdc3: Mm01243777_m1 (FAM); Mrpl19: Mm00452754_m1 (VIC); Thermo Fisher Scientific) in the second and third in vivo experiments to show that similar data can be obtained regardless of the real-time PCR method used. Data were normalized to the Mrpl19 reference gene level for presentation.

### 2.6. Plasma CK Measurement

Plasma fraction was separated by centrifugation of collected blood samples. Cygnus-auto CK (Shino-Test, Tokyo, Japan) was used to measure plasma creatine kinase levels using a Hitachi Automatic Analyzer Labospect 006 according to the Japan Society of Clinical Chemistry (JSCC, Tokyo, Japan) compatible method.

### 2.7. Immunohistochemistry

Gastrocnemius muscles were immediately fixed in 10% neutralized buffered formalin after being removed from euthanized animals and embedded in paraffin for histological analysis. Immunohistochemistry was carried out as described previously [21,22]. Paraffin sections were deparaffinized. For mouse IgG immunostaining, sections were incubated overnight with biotin-conjugated anti-mouse IgG goat polyclonal antibody (Vector Laboratories, Burlingame, CA, USA, Cat. No. BA-9200), followed by horseradish peroxidase-conjugated streptavidin (Dako, Glostrup, Denmark). For laminin immunostaining to stain the basal lamina underlying the myofiber plasma membrane, sections were incubated overnight with anti-laminin rabbit polyclonal antibody (Sigma-Aldrich, Cat. No. L9393-.2ML), followed by horseradish peroxidase-conjugated secondary antibody (anti-rabbit IgG goat polyclonal antibody; Nichirei Biosciences, Tokyo, Japan). The sections were colorized with 3,3′-diaminobenzidine (Nichirei Biosciences) and counterstained with hematoxylin.

The sections were photographed with an Aperio Scan Scope AT Turbo for image analysis (Leica Biosystems, Nussloch, Germany). ImageScope image analysis software (version 12, Leica Biosystems, Deer Park, IL, USA) was used to process images of more than 1000 muscle fibers from at least five different regions in each immunostained specimen. In IgG-immunostained sections, the numbers of IgG-immunopositive myofibers were manually counted. The number of centrally nucleated myofibers was manually counted in sections immunostained with laminin. The percentage of IgG-positive myofibers and centrally nucleated myofibers were determined.

The sections were photographed with a DP73 digital camera system (Olympus Corporation, Tokyo, Japan) equipped with a BX53 microscope (Olympus Corporation) for representative histological images.

### 2.8. Treadmill Running Test

A treadmill running test was performed as described previously [10,23]. The treadmill MK-690/RM (Muromachi Kikai, Tokyo, Japan) was set with a 7-degree uphill incline, and a 0.3 mA electric shock grid turned on. Mice were adapted for 5 min on the stationary treadmill belt. Running speed was initially set at 8 m/min for 5 min (adaptation running), then increased by 2 m/min every 2 min until exhaustion (consecutive contact to the shock grid for at least 4 s), or until the cut-off speed of 28 m/min.

### 2.9. Ex Vivo Muscle Force Test

Soleus muscles were immediately harvested from euthanized animals 13 or 14 days after TMX injection, and an ex vivo muscle force test was performed as previously described [10,24]. To summarize, both ends of the isolated soleus muscle-tendon were tied with silken threads to connect to the transducer. The soleus muscle was then mounted in the chamber of the skeletal muscle contraction system (Uchida Denshi, Hachioji, Japan). Muscles were electrically stimulated at the following setting: pulse rate 100 Hz; duration 0.1 ms; volts 50 V. Muscle contraction was recorded. The maximum tetanic force and the fatigue curve’s area under the curve (AUC) values were calculated using the Power Lab system (AD Instruments, Sydney, Australia) and LabChart Software (version 8, AD Instruments, Dunedin, New Zealand). The muscle contractile force was measured in a blinded manner.

### 2.10. Statistical Analysis

In vivo results were expressed as means ± S.E.M. For in vitro experiments, results were expressed as mean ± S.D. Statistical analyses were performed using SAS software (version 9, EXSUS, EPS Corporation, Tokyo, Japan). To compare two groups, differences between groups were calculated using the Student’s *t*-test and were considered statistically significant when two-sided *p* < 0.05 was obtained. To compare three or more groups, differences between groups were calculated using the Williams’ test and were considered statistically significant when one-sided *p* < 0.025 was obtained.

## 3. Results

### 3.1. MT-DUX4-ASO Decreased DUX4 and Its Target Gene Expression In Vitro (In Vitro Experiments)

**Figure 1:**
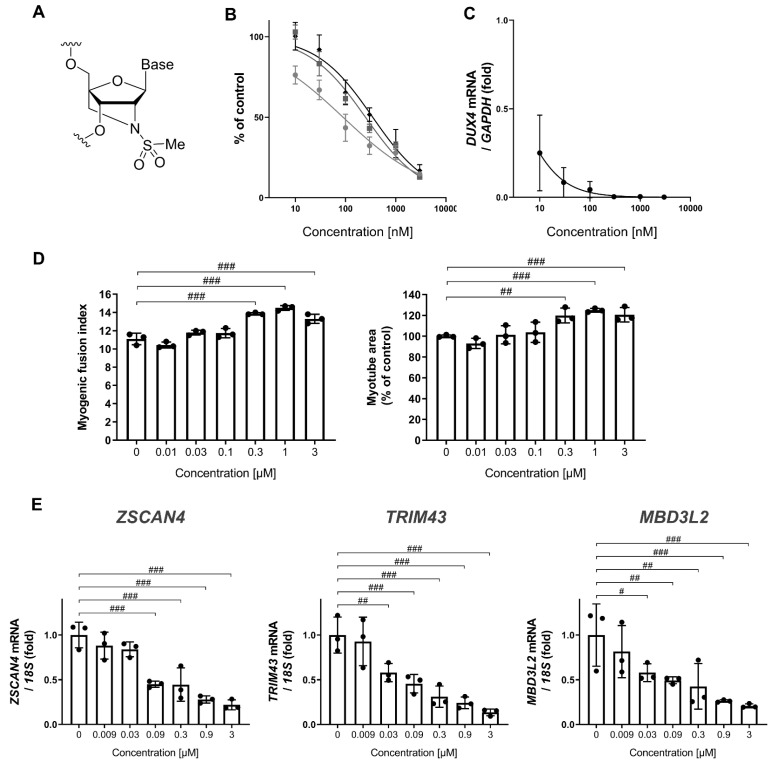
MT-DUX4-ASO significantly decreased the expression of DUX4 and its downstream genes in vitro. (**A**) The structure of 2′-*N*-methanesulfonyl-2′-amino-locked nucleic acid (ALNA[Ms]) used in MT-DUX4-ASO. (**B**) The effect of MT-DUX4-ASO on DUX4 expression level in DUX4 reporter gene assay using C2C12 cells. MT-DUX4-ASO was introduced into the cells by free uptake with the indicated concentration. Relative luciferase reporter activity from the mean level of vehicle (PBS)-treated cells is shown. Results from three independent experiments are shown in three different colors. (**C**) The effect of MT-DUX4-ASO on DUX4 gene expression in FSHD patient-derived myoblasts. MT-DUX4-ASO was introduced into the cells by free uptake with the indicated concentration. The expression level of the DUX4 genes is expressed as fold changes from the mean expression level of vehicle (PBS)-treated cells after normalization to the expression of reference gene. (**D**) The effect of MT-DUX4-ASO on the myotube formation of FSHD patient-derived myoblasts. MT-DUX4-ASO was introduced into the cells by free uptake with the indicated concentration. Myotube area is expressed as percent change from the mean level of vehicle (PBS)-treated cells. Myogenic fusion index was calculated as the percentage of the nuclei number in MHC-positive cells relative to the total nuclei number. (**E**) The effect of MT-DUX4-ASO on DUX4 target gene expression in FSHD patient-derived myoblasts. The expression levels of the DUX4 target genes are expressed as fold changes from the mean expression level of vehicle (PBS)-treated cells after normalization to the expression of reference gene. Data represent means ± S.D. Statistically significant differences are indicated by # (*p* < 0.025), ## (*p* < 0.005), and ### (*p* < 0.0005) (Williams’ test).

To investigate a disease-modifying drug for FSHD, we generated a novel gapmer ASO for the human DUX4 sequence, MT-DUX4-ASO, which was selected from the screening of several hundreds of candidates through in vitro and in vivo experiments such as those described below (data not included in this article). The resultant MT-DUX4-ASO has optimized ASO design including its sequence, which largely affects ASO’s in vivo activity. MT-DUX4-ASO uses 2′-*N*-methanesulfonyl-2′-amino-locked nucleic acid (ALNA[Ms]) (Figure 1A) [19] as artificial oligonucleotides. ALNA[Ms] is our original artificial nucleotide with high in vivo activity comparable to preceding locked nucleic acid (LNA) [19].

First, we evaluated the MT-DUX4-ASO’s potency in vitro. Exogenously expressed DUX4 causes cell death in vitro [25], leading to a decreased level of measured DUX4 mRNA, which hinders the accurate evaluation of the knockdown effect of ASO on DUX4 mRNA expression. Therefore, we used a reporter transgene system [26,27,28] containing a luciferase gene followed by a DUX4 open reading frame sequence inserted into the 3′-untranslated regions. MT-DUX4-ASO was introduced into C2C12 cells by free uptake (gymnosis) without transfection reagents. MT-DUX4-ASO reduced DUX4 expression with an IC_50_ value of 200 nM (Figure 1B). We also investigated the effect of MT-DUX4-ASO on DUX4 expression in FSHD patient-derived primary myoblasts in vitro [13,14,16]. MT-DUX4-ASO reduced DUX4 expression with an IC_50_ value of <10 nM by gymnotic treatment (Figure 1C).

We investigated the effect of MT-DUX4-ASO on impaired myotube formation in FSHD patient-derived myoblasts in vitro [13,14,16]. Although previous studies [13,14,16] used transfection reagents to enhance the intracellular uptake of treated ASOs, we introduced MT-DUX4-ASO into the cells by gymnosis without transfection reagents to recapitulate in vivo environment. MT-DUX4-ASO at 300 nM or more improved indicators of myotube formation, myogenic fusion index, and myotube area (*p* < 0.005; Williams’ test) (Figure 1D). In these FSHD myotubes, the expression levels of the human DUX4 target genes—ZSCAN4, TRIM43, and MBD3L2 [13,14,16]—were decreased by MT-DUX4-ASO with the IC_50_ values of 180 nM, 89 nM, and 108 nM, respectively (*p* < 0.025; Williams’ test) (Figure 1E), indicating its potency in patient muscle cells. DUX4 expression level was too low to be measured quantitatively in this myotube formation experiment, which requires a longer culture period of 15 days.

### 3.2. MT-DUX4-ASO Suppressed DUX4 Target Gene Expression in TMX-Untreated DUX4-TG Mice (In Vivo Experiment-1)

The ability of MT-DUX4-ASO treatment to suppress disease presentation in vivo was investigated using DUX4-TG mice [9,10]. In these mice, TMX injection induces skeletal-muscle-specific DUX4 expression, resulting in exacerbated muscular pathogenesis. However, DUX4-TG mice have mosaic DUX4 expression in a small subset of myofibers in skeletal muscle even without TMX induction [10,15], similar to the low level of DUX4 expression observed in FSHD patients’ skeletal muscles [7]. Therefore, we investigated the therapeutic effect of systemically administered MT-DUX4-ASO on the pathology induced by a low level of muscular DUX4 expression in TMX-untreated DUX4-TG mice.

**Figure 2:**
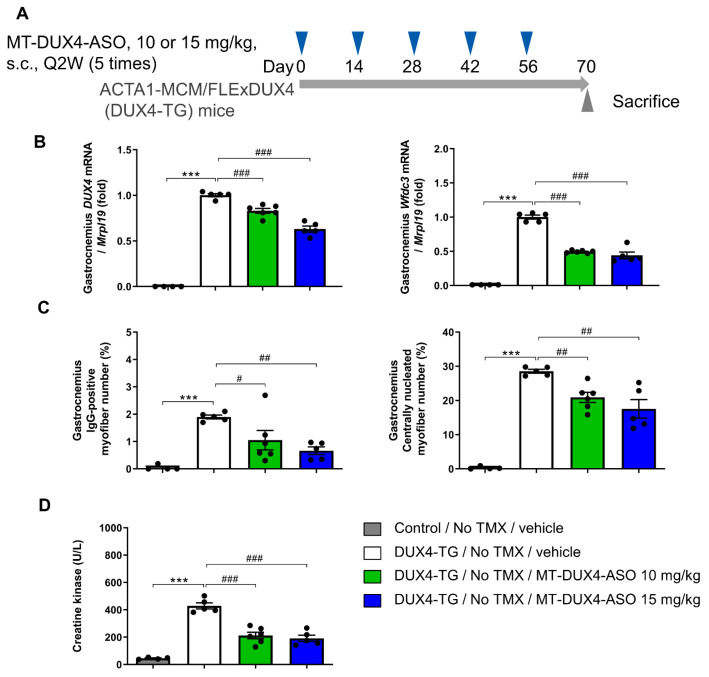
MT-DUX4-ASO improved muscle injury in TMX-untreated DUX4-TG mice. (**A**) Overview of the study. DUX4-TG mice were subcutaneously injected five times with 10 or 15 mg/kg of MT-DUX4-ASO or vehicle with an interval of two weeks (Q2W). Fourteen days after the final dose of MT-DUX4-ASO (day 70), animals were sacrificed, and gastrocnemius muscles were collected. (**B**) The effect of MT-DUX4-ASO on DUX4 and its target gene Wfdc3 expression in DUX4-TG gastrocnemius muscles. Data are expressed as fold changes from the mean expression level of vehicle-treated DUX4-TG muscles after normalization to the expression of reference gene Mrpl19. (**C**) The effect of MT-DUX4-ASO on the percentage of IgG-positive or centrally nucleated myofiber number in the histological analysis of gastrocnemius muscles in DUX4-TG mice. (**D**) The effect of MT-DUX4-ASO on muscle injury blood biomarker creatine kinase in DUX4-TG mice. Data represent means ± S.E.M. Statistically significant differences are indicated by *** (*p* < 0.001) (Student’s *t*-test), # (*p* < 0.025), ## (*p* < 0.005), and ### (*p* < 0.0005) (Williams’ test).

MT-DUX4-ASO at 10 or 15 mg/kg was subcutaneously administered five times every other week to TMX-untreated DUX4-TG mice. The mice were sacrificed two weeks after the last dose of MT-DUX4-ASO to examine whether MT-DUX4-ASO had a persistent effect during the two-week dosing intervals (Figure 2A). We examined the effect of MT-DUX4-ASO on DUX4 and its target gene expression in DUX4-TG muscles (Figure 2B). In the gastrocnemius muscles of DUX4-TG mice, DUX4 gene expression was increased compared with control mice (*p* < 0.001; Student’s *t*-test), although its expression was still low. DUX4 transgene expression is so low, and a poor measure, because there are so few DUX4-expressing cells, which are further reduced by DUX4-induced cell death [9,10]. DUX4 mRNA is also difficult to detect in FSHD muscle biopsies because of its low abundance [7]. The administration of 10 or 15 mg/kg of MT-DUX4-ASO reduced DUX4 gene expression in the DUX4-TG gastrocnemius muscles (*p* < 0.0005; Williams’ test).

Mouse DUX4 target gene Wfdc3 is reportedly a more accurate indicator of DUX4 expression levels [9,10]. While control gastrocnemius muscles had extremely low Wfdc3 gene expression, TMX-untreated DUX4-TG muscles had increased Wfdc3 gene expression due to leaky DUX4 misexpression (*p* < 0.001; Student’s *t*-test) (Figure 2B). Treatment with 10 or 15 mg/kg of MT-DUX4-ASO strongly reduced Wfdc3 gene expression in the gastrocnemius muscles by more than half (*p* < 0.0005; Williams’ test).

### 3.3. MT-DUX4-ASO Suppressed Muscle Injury in TMX-Untreated DUX4-TG Mice (In Vivo Experiment-1)

**Figure 3:**
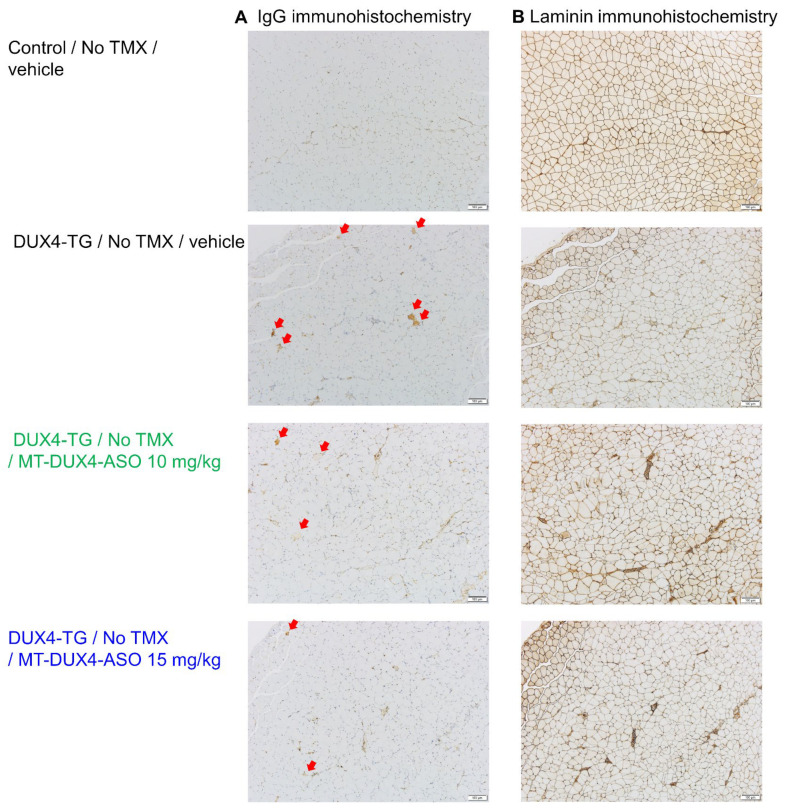
MT-DUX4-ASO improved histological muscle injury in TMX-untreated DUX4-TG mice. (**A**) Gastrocnemius muscles immunostained for dead myofiber marker IgG are shown. Red arrows indicate IgG-positive myofibers. (**B**) Laminin was immunostained in gastrocnemius muscles to label myofiber boundaries explicitly. Scale bars indicate 100 μm. See Figure 2C for the result of quantification of IgG-positive or centrally nucleated myofiber number.

In FSHD-affected skeletal muscles, only a subset of myofibers show DUX4-induced cell death [11,29]. Here, we used IgG immunostaining as a marker for dead myofibers to detect histological muscle injuries for preclinical drug efficacy studies in DUX4-TG mice, both sensitively and quantitatively, for the first time. Compromised plasma membrane integrity in dead myofibers allows intracellular deposition of serum proteins, including IgG [30]. Control mice’s gastrocnemius muscles had few IgG-positive myofibers (Figure 2C and Figure 3). Those TMX-untreated DUX4-TG mice with leaky DUX4 expression, on the other hand, showed a small increase in IgG-positive myofibers in a mosaic pattern (*p* < 0.001; Student’s *t*-test). Treatment with 10 mg/kg of MT-DUX4-ASO reduced the number of IgG-positive dead myofibers in the gastrocnemius muscles by almost half (*p* < 0.025; Williams’ test). Treatment with 15 mg/kg of MT-DUX4-ASO further decreased the number of IgG-positive myofibers (*p* < 0.005; Williams’ test).

We also studied the number of centrally nucleated myofibers found in gastrocnemius muscles in DUX4-TG mice [11,15,17,18,29]. The nuclei of regenerated mouse myofibers stay central for several months [31]. As a result, the number of centrally nucleated myofibers reflects the number of myofibers that died and regenerated over the previous several months. Muscle sections were immunostained with laminin to visualize myofiber outlines to study centrally nucleated myofiber numbers (Figure 3) [16,32]. Gastrocnemius muscles of control mice had few centrally nucleated myofibers (Figure 2C and Figure 3). Centrally nucleated myofibers were increased in DUX4-TG mice (*p* < 0.001; Student’s *t*-test). The MT-DUX4-ASO treatment at 10 or 15 mg/kg reduced the number of centrally nucleated myofibers (*p* < 0.005; Williams’ test), indicating that MT-DUX4-ASO had a sustained effect on muscle injuries.

Creatine kinase (CK), a blood skeletal muscle injury biomarker, is reportedly mildly elevated in FSHD patients and is correlated with disease severity [2,33]. However, due to its variability, blood CK testing is not required for the diagnosis of FSHD. Here, we have investigated blood CK levels of DUX4-TG mice for the first time (Figure 2D). Plasma CK levels in TMX-untreated DUX4-TG mice with leaky DUX4 expression were significantly higher than in control mice (*p* < 0.001; Student’s *t*-test). The administration of 10 or 15 mg/kg of MT-DUX4-ASO reduced plasma CK levels by more than half (*p* < 0.0005; Williams’ test).

### 3.4. MT-DUX4-ASO Prevented TMX-Induced DUX4 Target Gene Expression and Muscle Injury in DUX4-TG Mice (In Vivo Experiment-2)

Patients with FSHD have a mobility impairment, including difficulty ambulating, which has a significant impact on their daily lives [3,4,5]. Therefore, we next investigated the effect of MT-DUX4-ASO on motor function decline in DUX4-TG mice. We used TMX-treated DUX4-TG mice to study motor function because TMX-untreated DUX4-TG mice have an equivalent running ability to control mice in the treadmill test [10].

**Figure 4:**
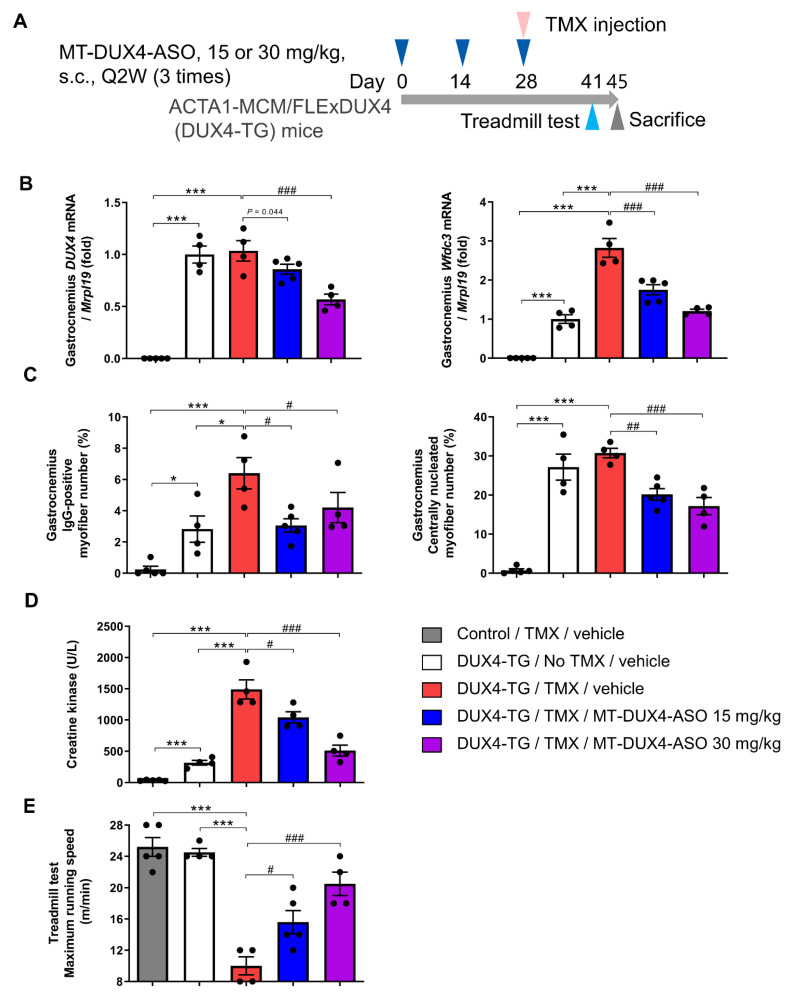
MT-DUX4-ASO prevented TMX-induced motor function decline in DUX4-TG mice. (**A**) Overview of the study. DUX4-TG mice were subcutaneously injected three times with 15 or 30 mg/kg of MT-DUX4-ASO or vehicle with an interval of two weeks (Q2W). On the day of the last dose of MT-DUX4-ASO (day 28), TMX was intraperitoneally injected to induce DUX4 expression. Thirteen days after the final dose of MT-DUX4-ASO (day 41), animals were run on a treadmill running test. Seventeen days after the final dose of MT-DUX4-ASO (day 45), animals were sacrificed, and gastrocnemius muscles were collected. (**B**) The effect of MT-DUX4-ASO on DUX4 and its target gene Wfdc3 expression in DUX4-TG gastrocnemius muscles. Data are expressed as fold changes from the mean expression level of TMX-untreated DUX4-TG muscles after normalization to the expression of reference gene Mrpl19. (**C**) The effect of MT-DUX4-ASO on the percentage of IgG-positive or centrally nucleated myofiber number in the histological analysis of gastrocnemius muscles in DUX4-TG mice. (**D**) The effect of MT-DUX4-ASO on muscle injury blood biomarker creatine kinase in DUX4-TG mice. (**E**) The effect of MT-DUX4-ASO on the maximum running speed of DUX4-TG mice in treadmill running test. Running speed was initially set at 8 m/min and increased by 2 m/min every 2 min. Mice were run until consecutive contact with the electric shock grid or up to the cut-off speed of 28 m/min. Data represent means ± S.E.M. Statistically significant differences are indicated by * (*p* < 0.05), *** (*p* < 0.001) (Student’s *t*-test), # (*p* < 0.025), ## (*p* < 0.005), and ### (*p* < 0.0005) (Williams’ test).

MT-DUX4-ASO at 15 or 30 mg/kg was subcutaneously administered three times at two-week intervals to DUX4-TG mice, and pathogenesis was induced by TMX injection on the last day of MT-DUX4-ASO administration (Figure 4A). The mice were sacrificed two weeks after the last dose of MT-DUX4-ASO.

TMX injection to DUX4-TG mice did not significantly increase DUX4 gene expression in gastrocnemius muscles, probably because the low level of DUX4 transgene expression is a poor measure as described above [9,10]. Nevertheless, MT-DUX4-ASO at 30 mg/kg reduced DUX4 gene expression in the gastrocnemius muscles by nearly half (*p* < 0.0005; Williams’ test) (Figure 4B). Treatment with 15 mg/kg of MT-DUX4-ASO reduced the mean value of DUX4 gene expression in TMX-treated DUX4-TG mice, but the difference was not statistically significant (*p* = 0.044, Williams’ test).

TMX injection to DUX4-TG mice increased the expression of DUX4 target gene Wfdc3, a more accurate indicator of DUX4 expression levels (*p* < 0.001; Student’s *t*-test) (Figure 4B), as reported previously [9,10]. The administration of 15 mg/kg of MT-DUX4-ASO strongly reduced Wfdc3 gene expression in the muscles (*p* < 0.0005; Williams’ test). The administration of 30 mg/kg of MT-DUX4-ASO further decreased the Wfdc3 expression level (*p* < 0.0005; Williams’ test).

**Figure 5:**
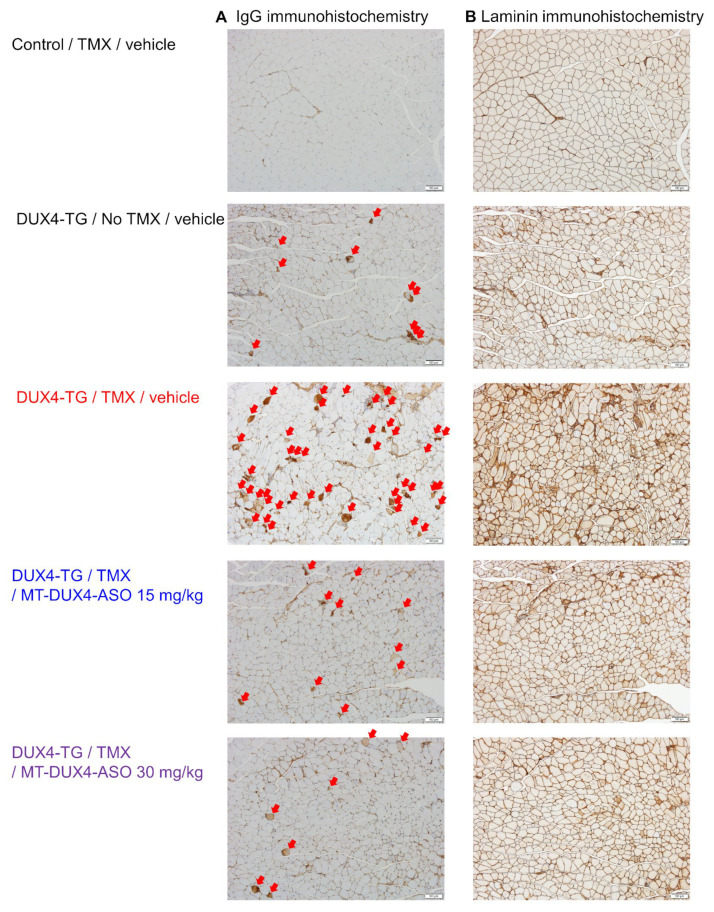
MT-DUX4-ASO improved histological muscle injury in TMX-treated DUX4-TG mice. (**A**) Gastrocnemius muscles immunostained for dead myofiber marker IgG are shown. Red arrows indicate IgG-positive myofibers. (**B**) Laminin was immunostained in gastrocnemius muscles to label myofiber boundaries explicitly. Scale bars indicate 100 μm. See Figure 4C for the result of the quantification of IgG-positive or centrally nucleated myofiber number.

Next, we evaluated the effect of MT-DUX4-ASO on histological muscular injuries (Figure 4C and Figure 5). TMX-untreated DUX4-TG mice showed an increase in the number of IgG-positive dead myofibers (*p* < 0.05; Student’s *t*-test), while TMX injection further increased it (*p* < 0.05; Student’s *t*-test). Treatment with 15 or 30 mg/kg MT-DUX4-ASO reduced the number of IgG-positive myofibers in the gastrocnemius muscles (*p* < 0.025; Williams’ test).

We also looked at the number of centrally nucleated myofibers as a marker of muscular injuries in the previous several months (Figure 4C and Figure 5). The gastrocnemius of TMX-untreated DUX4-TG mice with leaky DUX4 expression showed an increase in the number of centrally nucleated myofibers (*p* < 0.001; Student’s *t*-test). Interestingly, TMX injection yielded no significant difference in the central nuclei, possibly because centrally nucleated myofibers had not regenerated much when TMX-induced muscle cell death was at its peak [10]. The MT-DUX4-ASO treatment at 15 or 30 mg/kg strongly reduced the number of centrally nucleated myofibers (*p* < 0.005; Williams’ test), indicating MT-DUX4-ASO’s persistent effect on muscle injuries.

We also investigated the levels of blood muscle injury biomarker, CK, in TMX-treated DUX4-TG mice (Figure 4D). TMX injection markedly accelerated the increase in the plasma CK level in DUX4-TG mice (*p* < 0.001; Student’s *t*-test). The administration of 15 mg/kg of MT-DUX4-ASO reduced plasma CK levels (*p* < 0.025; Williams’ test). Notably, 30 mg/kg of MT-DUX4-ASO further lowered the plasma CK level to a level close to that of TMX-untreated DUX4-TG mice (*p* < 0.0005; Williams’ test).

### 3.5. MT-DUX4-ASO Prevented TMX-Induced Motor Function Decline in DUX4-TG Mice (In Vivo Experiment-2)

We have investigated the effect of MT-DUX4-ASO on motor function decline in TMX-treated DUX4-TG mice using a treadmill motor function test [10]. The mice were forced to run to exhaustion over a running belt at a gradually increasing speed in the test. TMX-untreated DUX4-TG mice could run as fast as the control mice [10]. As previously reported [10], TMX-induced systemic muscular DUX4 expression severely reduced the maximum running speed of DUX4-TG mice 13 days after TMX injection (*p* < 0.001; Student’s *t*-test) (Figure 4E). The administration of 15 mg/kg of MT-DUX4-ASO prevented the TMX-induced decline in the maximum running speed (*p* < 0.025; Williams’ test). Notably, 30 mg/kg of MT-DUX4-ASO improved the maximum running speed of TMX-treated DUX4-TG mice to a level close to that of control mice or TMX-untreated DUX4-TG mice (*p* < 0.0005; Williams’ test).

### 3.6. MT-DUX4-ASO Prevented TMX-Induced Decline in Muscle Force and Motor Function in DUX4-TG Mice with a Lower Dose of 10 mg/kg (In Vivo Experiment-3)

**Figure 6:**
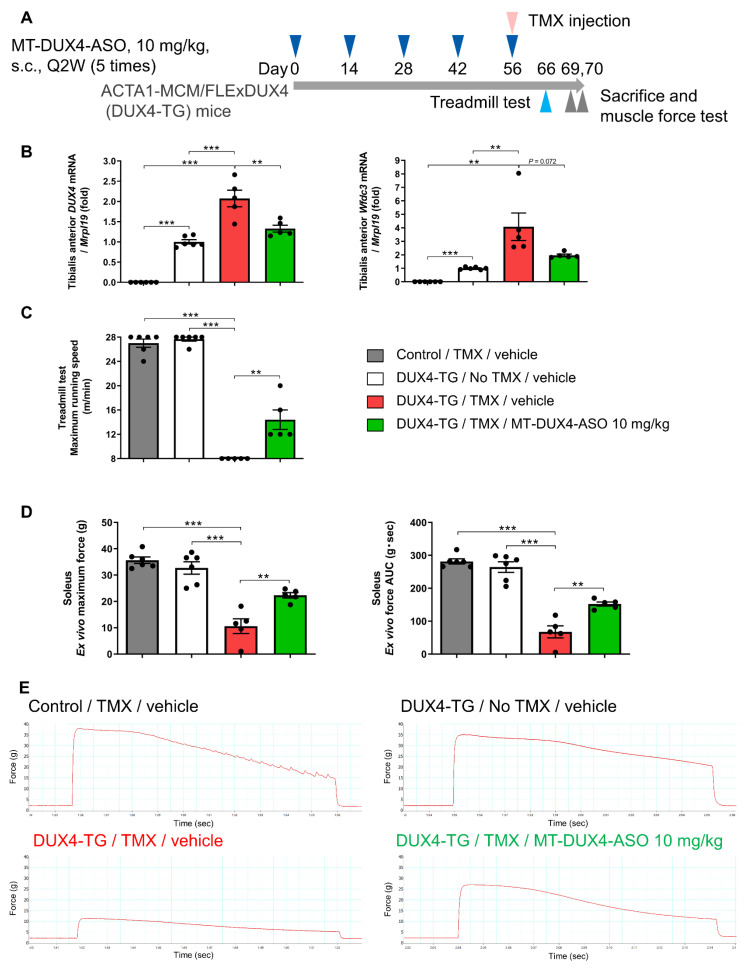
MT-DUX4-ASO prevented TMX-induced decline in motor function and muscle force in DUX4-TG mice. (**A**) Overview of the study. DUX4-TG mice were subcutaneously injected five times with 10 mg/kg of MT-DUX4-ASO or vehicle with an interval of two weeks (Q2W). On the day of the last dose of MT-DUX4-ASO (day 56), TMX was intraperitoneally injected to induce DUX4 expression. Ten days after the final dose of MT-DUX4-ASO (day 66), animals were run on a treadmill running test. Thirteen or fourteen days after the final dose of MT-DUX4-ASO (day 69 or 70), animals were sacrificed, and soleus muscles were harvested and subjected to an ex vivo muscle force test. Tibialis anterior muscles were also collected. (**B**) The effect of 10 mg/kg of MT-DUX4-ASO on DUX4 and its target gene Wfdc3 expression in DUX4-TG tibialis anterior muscles. Data are expressed as fold changes from the mean expression level of TMX-untreated DUX4-TG muscles after normalization to the expression of reference gene Mrpl19. (**C**) The effect of 10 mg/kg of MT-DUX4-ASO on the maximum running speed of DUX4-TG mice in treadmill running test. Running speed was initially set at 8 m/min and increased by 2 m/min every 2 min. Mice were run until consecutive contact with the electric shock grid or up to the cut-off speed of 28 m/min. (**D**) The effect of 10 mg/kg of MT-DUX4-ASO on ex vivo soleus muscle force in DUX4-TG mice. The maximum tetanic force and area under curve (AUC) values of the fatigue curve of the collected soleus muscles are shown. (**E**) The representative fatigue curve from each test group in ex vivo soleus muscle force test is shown. Data represent means ± S.E.M. Statistically significant differences are indicated by ** (*p* < 0.01), and *** (*p* < 0.001) (Student’s *t*-test).

We also investigated the effect of MT-DUX4-ASO on motor function and muscle force with a lower dose of 10 mg/kg subcutaneously administered five times at two-week intervals (Figure 6A). TMX injection upregulated the expression of DUX4 and its target gene Wfdc3 in tibialis anterior muscles (*p* < 0.01; Student’s *t*-test) (Figure 6B). The administration of 10 mg/kg of MT-DUX4-ASO significantly decreased DUX4 gene expression (*p* < 0.01; Student’s *t*-test). The administration of 10 mg/kg of MT-DUX4-ASO decreased Wfdc3 gene expression, which was not statistically significant (*p* = 0.072, Student’s *t*-test) because the variability of the values was different between the groups.

TMX-treated DUX4-TG mice showed a dramatic decrease in maximum running speed in the treadmill running test ten days after TMX injection, as described above (*p* < 0.001; Student’s *t*-test) (Figure 6C). Administration of 10 mg/kg of MT-DUX4-ASO significantly prevented the TMX-induced decrease in DUX4-TG mice’s maximum running speed (*p* < 0.01; Student’s *t*-test).

To corroborate the result of the treadmill motor function test, we also investigated the effect of MT-DUX4-ASO on the ex vivo soleus muscle force test (Figure 6D,E). The soleus muscles isolated from TMX-untreated DUX4-TG mice demonstrated a muscle force comparable to that of control mice. TMX injection markedly decreased soleus muscle force in DUX4-TG mice (*p* < 0.001; Student’s *t*-test). Treatment with 10 mg/kg of MT-DUX4-ASO significantly prevented the TMX-induced muscle force decline (*p* < 0.01; Student’s *t*-test), which was consistent with the results of the treadmill running test.

The overview of the three in vivo efficacy experiments is summarized in Table 1.

### 3.7. MT-DUX4-ASO at 100 mg/kg Was Well Tolerated in Mice (Tolerability Experiment)

We exploratorily investigated the tolerability of MT-DUX4-ASO in mice. Intravenous MT-DUX4-ASO at 100 mg/kg was administered once daily for 4 days to normal mice. The blood was collected three days after the last dose of MT-DUX4-ASO. MT-DUX4-ASO had no significant effect on liver injury marker (AST, ALT) or kidney injury marker (urea nitrogen, creatinine) levels in serum, indicating that MT-DUX4-ASO was well tolerated at a high daily dose of 100 mg/kg in mice (Table 2) (Student’s *t*-test).

## 4. Discussion

An effective treatment to improve the disease in FSHD patients has been long awaited. In this study, we found that a novel MT-DUX4-ASO is highly effective at preventing motor function deterioration and muscle weakness, implying its potential to improve pathology in FSHD patients. In addition, we used histological and blood biomarkers for muscle injury in an FSHD preclinical efficacy study for the first time. The use of these markers might allow quantitative and rapid assessments of muscle injuries in preclinical drug efficacy studies for FSHD.

In this study, we have used IgG immunostaining and central nuclei to quantitate pathological changes in FSHD model mice. Muscle biopsies from FSHD patients show both necrosis and central nuclei [11,29]. While the IgG-positive dead myofiber number shows current muscle injury, the central nuclei number shows muscle injuries that have occurred in the past several months [31]. Interestingly, MT-DUX4-ASO treatment improved both parameters in DUX4-TG mice, suggesting its sustained effect even when administered every other week.

Blood CK is a well-known skeletal muscle injury marker and is reportedly elevated in patients with FSHD [2,33]. However, blood CK has not previously been used in the preclinical study of FSHD model mice. In this study, we looked at blood CK levels in DUX4-TG mice and discovered that blood CK was elevated as TMX injection exacerbated muscle injury. MT-DUX4-ASO treatment significantly reduced the blood CK levels in DUX4-TG mice. The use of a blood biomarker could facilitate preclinical drug efficacy studies because these markers can be easily measured. Although we managed to quantify low DUX4 gene expression in DUX4-TG muscles, DUX4 expression is quite difficult to detect in FSHD-patient-derived muscle biopsies due to its fairly low expression [7], which makes the clinical evaluation of ASOs in FSHD patients difficult. The development of blood biomarkers for FSHD would facilitate clinical trials, especially in the early phase.

TMX-untreated DUX4-TG mice have leaky DUX4 expression in only a small subset of myofibers [10], which recapitulates the DUX4 expression in only a small fraction of muscle cells in FSHD patients [7]. Therefore, we have studied the effect of MT-DUX4-ASO on muscular DUX4 target gene expression and muscular injuries in TMX-untreated DUX4-TG mice. However, we found no significant decline in the treadmill running performance of TMX-untreated DUX4-TG mice compared with that of control mice (Figure 4E and Figure 6C) as described previously [10], although Bouwman et al. [15] showed the decline of treadmill test running time in TMX-untreated DUX4-TG mice. TMX injection causes transient symptoms with a 2-week peak, probably because dying DUX4-expressing muscle cells are compensated by regenerating non-DUX4-expressing muscle cells [10]. Lu-Nguyen et al. [17] have demonstrated the chronic disease progression model, where DUX4-TG mice were repeatedly treated with 1.5 mg/kg of TMX every other week. However, they did not show how much the disease was exacerbated by such a low dose of TMX compared with TMX-untreated DUX4-TG mice. On the other hand, repeated injection of TMX at a higher dose can easily become lethal [10,17]. We need further fine refinement to establish the test conditions to study the benefit of MT-DUX4-ASO for long-term motor function deficits in DUX4-TG mice. Thus, in this study, we used DUX4-TG mice with a single TMX injection to investigate the effect of MT-DUX4-ASO on the motor function.

In this study, MT-DUX4-ASO was administered three or five times at two-week intervals to DUX4-TG mice, and pathogenesis was induced by TMX injection on the last day of MT-DUX4-ASO administration. The mice were sacrificed two weeks after the last dose of MT-DUX4-ASO. We have used this study regimen because of two reasons. First, we investigated the MT-DUX4-ASO’s effect two weeks after the last dose to confirm its sustained effect during the two-week dosing intervals. Because the disease induced by TMX is acute and transient with its peak of 2 weeks [10], we injected TMX two weeks before the timing of the investigation in order to investigate ASO’s effect when the disease induced by TMX was at its peak. Second, MT-DUX4-ASO was dosed three or five times before TMX-induced disease exacerbation because, to exert its effect, ASO needs to be sufficiently accumulated in skeletal muscles by repeated dosing when systemically administered at clinically applicable low doses [34,35].

We have demonstrated that MT-DUX4-ASO inhibited the muscle injury induced by a leaky low level of DUX4 expression in TMX-untreated DUX4-TG mice, which recapitulates fairly low DUX4 expression in FSHD-affected muscles [7]. On the other hand, TMX-induced exacerbation in DUX4-TG mice mimics intermittent bursts of muscular DUX4 expression in FSHD patient muscles, which results in stochastic active dystrophic lesions and subsequent irreversible changes [11,12]. Therefore, the preventive effect of MT-DUX4-ASO in TMX-treated DUX4-TG mice indicates its potential to prevent the emergence of active muscle lesions and, thus, symptomatic disease progression, including mobility impairment in FSHD patients when these patients are regularly treated with MT-DUX4-ASO.

Even though several ASOs have emerged as therapies for muscular diseases, there are currently no effective drugs, including ASOs, available for FSHD. Only a few groups have reported that systemic administration of ASOs conjugated with ligands for delivery to skeletal muscles improved the pathology of DUX4-TG mice [15,16,17,18]. Lim et al. [13,14] showed the suppression of muscular DUX4 gene expression in TMX-untreated DUX4-TG mice by the local intramuscular administration of unconjugated gapmer ASOs. Bouwman et al. [15] demonstrated a significant improvement in TMX-untreated DUX4-TG mice by subcutaneous administration of 50 mg/kg of palmitoyl-conjugated antisense gapmer once or twice a week, which is a dose 10–20 times higher than MT-DUX4-ASO, although their gapmer has palmitoyl cell-penetrating conjugation. Lu-Nguyen et al. [16,17,18] demonstrated that intraperitoneal administration of cell-penetrating octaguanidine-dendrimer-conjugated phosphorodiamidate morpholino oligomers ASO improved the pathology of TMX-treated DUX4-TG mice, although the intraperitoneal administration route is not feasible for clinical use. Both groups used conjugated ASOs to help nonspecifically deliver the systemically administered ASOs into cells. To manufacture these conjugations for clinical use, a complicated production process and a higher cost would be required. We demonstrated that, despite the lack of conjugation to a ligand for drug delivery, the optimized ASO, MT-DUX4-ASO, could significantly improve muscle injury and motor function by subcutaneously administering as little as 10 mg/kg every other week. A highly optimized ASO design of MT-DUX4-ASO selected from several hundred candidates would have enabled its high in vivo activity. Indeed, MT-DUX4-ASO could effectively decrease the expression of DUX4 target genes and improve the reduced myogenic differentiation in in vitro FSHD myotubes, even without transfection reagents, which suggests the high potency of MT-DUX4-ASO. In addition to MT-DUX4-ASO’s high potency, the enhanced delivery to injured and regenerating “leaky” myofibers with increased membrane permeability in DUX4-TG mice with CK elevation might facilitate the effect of unconjugated ASO [34]. Indeed, unconjugated ASOs are in clinical use for the treatment of Duchenne muscular dystrophy where affected muscles show increased membrane permeability with marked CK elevation [36].

## 5. Conclusions

In this study, we investigated a novel gapmer ASO that decreased DUX4 and its target genes in FSHD patient-derived myoblasts in vitro. We showed for the first time that a systemically administered ASO lacking a ligand for drug delivery significantly improved muscle injuries in TMX-untreated DUX4-TG mice and prevented muscle injuries and motor impairment in TMX-treated DUX4-TG mice. Importantly, despite being a cost-competitive unconjugated ASO, MT-DUX4-ASO achieved these effects with a clinically feasible dosing regimen. The subcutaneous administration route and two-week dosing interval of MT-DUX4-ASO will be quite feasible and convenient for clinical use by FSHD patients who require life-long treatment because DUX4 expression occurs intermittently and randomly throughout their life in only a subset of myofibers at a time.

## Figures and Tables

**Table 1:** Overview of the three in vivo efficacy experiments using DUX4-TG mice.

Exp.	Mouse Sex	TMX-Treated	MT-DUX4-ASO, Dosing	Investigated Changes
1	Female	Untreated	10 or 15 mg/kg, s.c., Q2W, 5 times	Muscular gene expression, and muscle injuries (histology and plasma CK)
2	Female	5 mg/kg, i.*p*.	15 or 30 mg/kg, s.c., Q2W, 3 times	Muscular gene expression, muscle injuries (histology and plasma CK), and motor function (treadmill test)
3	Male	7.5 mg/kg, i.*p*.	10 mg/kg, s.c., Q2W, 5 times	Muscular gene expression, and motor function (treadmill test and muscle force)

Q2W: dosing interval of two weeks.

**Table 2:** MT-DUX4-ASO at a high dose was well tolerated in normal mice.

	Vehicle	MT-DUX4-ASO, 100 mg/kg, i.v., QDx4
AST (U/L)	53.8 ± 2.5	60.2 ± 3.3
ALT (U/L)	30.4 ± 3.6	38.6 ± 3.7
Urea nitrogen (mg/dL)	20.34 ± 1.17	18.62 ± 1.64
Creatinine (mg/dL)	0.056 ± 0.004	0.054 ± 0.002

Male normal (Crl:CD1(ICR)) mice were administered once daily (QD) with 100 mg/kg of intravenous MT-DUX4-ASO or vehicle for 4 days from 6 weeks of age. Three days after the final dose of MT-DUX4-ASO, animals were sacrificed, and blood was collected. The effect of MT-DUX4-ASO on blood chemistry was examined. Data represent means ± S.E.M. Statistically significant differences were not found between groups (Student’s *t*-test). AST, aspartate aminotransferase; ALT, alanine aminotransferase.

## Data Availability

Not applicable.
